# Metagenomic Profile of the Viral Communities in *Rhipicephalus* spp. Ticks from Yunnan, China

**DOI:** 10.1371/journal.pone.0121609

**Published:** 2015-03-23

**Authors:** Han Xia, Cheng Hu, Dake Zhang, Shuang Tang, Zhong Zhang, Zheng Kou, Zhaojun Fan, Dennis Bente, Changqing Zeng, Tianxian Li

**Affiliations:** 1 State Key Laboratory of Virology, Wuhan Institute of Virology, Chinese Academy of Sciences, Wuhan, Hubei, China; 2 Department of Microbiology and Immunology, University of Texas Medical Branch, Galveston, TX, United States of America; 3 International School of Software, Wuhan University, Wuhan, Hubei, China; 4 Laboratory of Genome Variations and Precision Biomedicine, Beijing Institute of Genomics, Chinese Academy of Sciences. NO.1 Beichen West Road, Chaoyang District, Beijing, China; Metabiota, UNITED STATES

## Abstract

Besides mosquitoes, ticks are regarded as the primary source of vector-borne infectious diseases. Indeed, a wide variety of severe infectious human diseases, including those involving viruses, are transmitted by ticks in many parts of the world. To date, there are no published reports on the use of next-generation sequencing for studying viral diversity in ticks or discovering new viruses in these arthropods from China. Here, Ion-torrent sequencing was used to investigate the presence of viruses in three *Rhipicephalus* spp. tick pools (NY-11, NY-13, and MM-13) collected from the Menglian district of Yunnan, China. The sequencing run resulted in 3,641,088, 3,106,733, and 3,871,851 reads in each tick pool after trimming. Reads and assembled contiguous sequences (contigs) were subject to basic local alignment search tool analysis against the GenBank database. Large numbers of reads and contigs related to known viral sequences corresponding to a broad range of viral families were identified. Some of the sequences originated from viruses that have not been described previously in ticks. Our findings will facilitate better understanding of the tick virome, and add to our current knowledge of disease-causing viruses in ticks living under natural conditions.

## Introduction

Ticks are second only to mosquitoes as important arthropod vectors for spreading viruses from wildlife to domestic animals and humans. They are also a source of unknown viruses. To date, at least 38 known viral species are transmitted by ticks, and some of them are a significant threat to human health [1]. Such viruses include tick-borne encephalitis virus [2], Crimean-Congo hemorrhagic fever virus (CCHFV) [3], Kyasanur forest disease virus [4], Alkhurma virus, severe fever with thrombocytopenia syndrome virus (SFTSV) [1, 5], and Heartland virus (HRTV)[6]. Additionally, several tick-borne viruses also threaten the health of livestock; these include Africa swine fever virus, Nairobi sheep disease virus (NSDV), and louping ill virus [7, 8]. Among ixodid ticks, viral disease-causing vectors are found mostly in the following genera: *Ixodes*, *Haemaphysalis*, *Hyalomma*, *Amblyomma*, *Dermacentor*, *Rhipicephalus*, *and Boophilus* [1].

In recent years, novel tick-borne viral diseases have emerged worldwide. From 2009 to 2011, an acute febrile illness of tick-borne origin was noted in several Chinese provinces and killed about 30% of the people infected. The novel virus, SFTSV, which belongs to the *Phlebovirus* genus of the family *Bunyaviridae*, was found to be the causative agent of severe fever with thrombocytopenia syndrome. Later on, *Haemaphysalis longicornis* was identified as the primary vector of SFTSV [5]. HRTV, another novel tick-borne phlebovirus associated with two cases of critical febrile illness in humans, was found in the United States in 2009. *Amblyomma americanum* ticks have been suggested as potential vectors of this disease [6, 9].

Next generation sequencing (NGS) technologies provide a powerful means of studying viral metagenomics, and can help us to gain better understanding of viral populations and discover unknown viruses in a number of environments [10, 11]. NGS is especially useful for assessment of uncultured samples, such as feces, blood, water, air and potential viral reservoirs [12–17]. The study which used NGS to explore the viral community in mosquitoes presented that mosquito virome contained sequences related to a broad range of animal, plant, insect and bacterial viruses. And the majority of the sequences from viral community in mosquitoes were novel [16]. Recently a research about tick virome from *Amblyomma americanum*, *Dermacentor variablilits*, and *Ixodes scapularis* ticks in the United States was published. Their results reveal novel highly divergent viruses in ticks, which include nairoviruses, phleboviruses, monoegavirusand viruses with similarity to plant and insect viruses [17]. However, there are no published studies on the use of NGS for exploring the viral diversity present in ticks from China.

Recent reports indicate that many tick-borne diseases exist in the Yunnan Province of China, such as Kyasanur forest disease, Crimean-Congo hemorrhagic fever, Colorado tick fever, and severe fever with thrombocytopenia syndrome [18–20]. The Menglian district, which belongs to subtropical lower mountainous areas, is located in southwest Yunnan close to the borders of China and Laos and China and Myanmar. The climate in Menglian is typically subtropical, warm and moist, and the abundant plants and wild animals (e.g., bearcat, deer, hare, mouse, and monkey) in this region create a suitable habitat for ticks. Additionally, many residents depend on raising livestock (e.g., goat, dog, cattle, buffalo, and horse), planting tobacco or tea for their economic livelihoods, and some even share their houses with livestock. These life habits may increase the risk of infection with tick-borne diseases from tick bites through close contact with livestock and plantations. An epidemiological study in the southeast region of Yunnan in 2008 revealed that partial sequences related to the CCHFV S segment were detected in some tick samples [21]. *Rhipicephalus* is known to associate with many viral pathogens, such as Thogoto virus (*Orthomyxoviridae*, genus *Thogoto*), Wad Medani virus (*Reoviridae*, genus *Orbivirus*), CCHFV and NSDV (*Bunyaviridae*, genus *Nairovirus*), Kismayo virus and Chim virus (*Bunyaviridae*, genus *Phlebovirus*), and Kandam virus (*Flaviviridae*, genus *Flavivirus*), all of which can cause disease in livestock and humans [1]. *Rhipicephalus* is the one of the most common tick genus in Menglian and generally throughout southwest China [22, 23], which preferably feeds on livestock and wild animals. The *Rhipicephalus microplus*, *R*. *haemaphysaloides* and *R*. *sanguineus* are very common tick species in this region.

In this study, Ion-torrent sequencing was used to investigate the presence of viruses in *Rhipicephalus* spp. ticks collected from the field in the Menglian district of Yunnan, China. The viral communities from three tick pools from two collection sites were analyzed and compared, and numerous virus-related sequences were identified.

## Methods

### Sample collection and taxonomy identification

**Fig 1:**
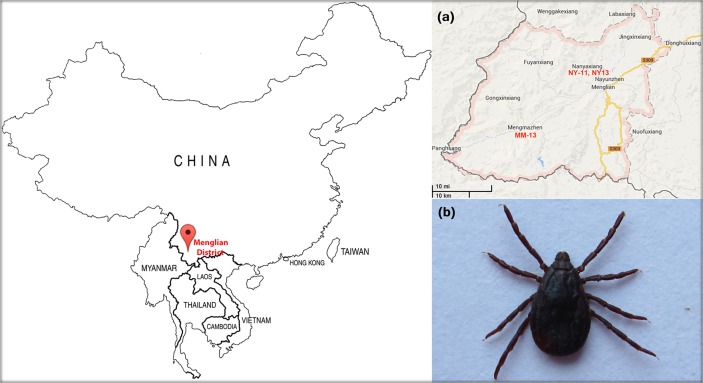
Tick species and sample collection map. *(a)* In the Menglian district, tick pools NY-11 and NY-13 were collected from Nayun, while MM-13 was collected from Mengma *(b)* A tick representing the Rhipicephalus genus from this region.Ticks were collected by the Menglian Center for Disease Control and Prevention (China) using a standard dragging method.

A total of 387 ticks were collected in the Menglian district of Yunnan, China in 2011 and 2013 (Fig. 1). One-hundred and twenty-seven of them were collected from Nayun (Latitude: 22.3, Longitude: 99.5, Altitude: 1143 m) in May, 2011 (NY-11), 150 from Nayun in May, 2013 (NY-13), and 110 from Mengma (Latitude: 22.8, Longitude: 100.9, Altitude: 1301 m) in May, 2013 (MM-13). No specific permissions for tick collection were required in these locations.

Ticks were stored in tubes and transported to the laboratory immediately. Morphological and molecular identification were used for tick taxonomic classification. The primers ITS-1 and ITS-2 (S1 Table), which were used for molecular identification, target the internal transcribed spacers of ribosomal DNA [24]. From each group, we selected 20 adult unfed *Rhipicephalus* spp. ticks as a pool for Ion-torrent sequencing (Fig. 1 (B)). The remaining *Rhipicephalus* spp. ticks in each group were stored at −80°C. To prevent contamination, the ticks used for Ion-torrent sequencing were surface-sterilized with 3% hydrogen peroxide, followed by 95% ethanol and 1M sodium hypochlorite. Ticks treated in this manner were washed and preserved in PBS before viral nucleic acids were extracted.

### Viral nucleic acid extraction

Each tick pool was homogenized through Tissuelyser II (Qiagen, Germany), 30 cycles/sec with 1 ml of phosphate-buffered saline and 5 mm stainless steel beads, and then then centrifuged at 5,000 × g for 10 min. The supernatant was transferred to a fresh tube and then centrifuged at 13,000 × g for 15 min. The supernatant was filtered through a 0.45 μM Millex filter (Millipore, USA) to remove eukaryotic and bacterial cell-sized particles. Total viral nucleic acids (DNA and RNA) were extracted from a 140-μl sample (from the above step) using a QIAamp Viral RNA Mini kit (Qiagen, Germany) according to the manufacturer’s instructions. Nucleic acids were eluted into 40 μl of AVE buffer [13].

### cDNA library preparation for Ion-torrent sequencing

First-strand cDNA synthesis was performed with a SuperScript III First-Strand System Kit (Invitrogen, USA). An 8 μl volume of purified viral nucleic acids from each tick pool was mixed with 1 μl of 10 mM deoxynucleoside triphosphate and 1 μl (50 ng/μl) of Brs primer (a random primer with the tag sequence at the 5’ end) (S1 Table), after which the solution was denatured at 65°C for 5 min, and placed on ice for 1 min. A 10 μl aliquot of the cDNA synthesis mix containing 2 μl of 10 × RT buffer, 4 μl of 25 mM MgCl_2_, 2 μl of 0.1 M DTT, 1 μl of RNaseOUT(40 U/μl) and 1 μl of SuperScript III RT (200 U/μl) was added to each nucleic acid–primer mixture. The reaction mixture was incubated at 25°C for 10 min and 50°C for 50 min, followed by enzyme inactivation at 85°C for 5 min and chilling on ice for 1 min. For the second-strand cDNA synthesis, 0.5 μl (20 pmol) of Brs primer, 2.5 μl of 10 × Klenow fragment buffer, and 2 μl of Klenow fragment (3.5 U/ μl, Takara, Japan) were added. The reaction mixture was incubated at 25°C for 10 min and 37°C for 60 min, followed by enzyme inactivation at 75°C for 10 min [13].

PCR assays were performed using a primer identical to the tag sequence of the Bra primer and using Phusion High-Fidelity DNA polymerase (New England Biolabs, USA), and lower cycles (≤20 cycles) were used to reducing amplification bias. Next, the products were purified to acquire three cDNA libraries. Each cDNA library was loaded onto one single Ion-Torrent 318 chip, and Ion-Torrent sequencing (Life Technologies, USA) was performed by the Beijing Institute of Genomics at the Chinese Academy of Sciences.

### Bioinformatics

Raw data were filtered into short (<100 bp) and long (>350 bp) reads, low quality scores (<20), exact duplicate reads and trimmed primer sequences by PRINSEQ 0.20.4 (http://edwards.sdsu.edu/cgi-bin/prinseq/prinseq.cgi) and Mothur v.1.33.0 (http://www.mothur.org/). The metagenomic reads were subjected to a basic local alignment search tool (BLASTx) comparison against the National Center for Biotechnology Information (NCBI, http://www.ncbi.nlm.nih.gov/) non-redundant database (nr) and its viral database by using an E-value cutoff of 1e-04. The taxonomy of the viral community was analyzed and compared using the MEGAN5 analysis toolset (http://ab.inf.uni-tuebingen.de/software/megan/). The reads that hit viral database sequences were assembled by a metagenomic *de novo* assembly for the Ion-Torrent model of SeqMan NGen 11.2.1 (DNASTAR, http://www.dnastar.com/), and the contiguous sequences (contigs) obtained were subject to BLAST against GenBank using BLASTn and BLASTx.

### PCR screening

cDNA from tick pools NY-11, NY-13 and MM-13 were amplified and then subjected to PCR with specific primers based on the sequence-selected contigs from the tick viromes (S1 Table). The PCR products were sequenced to verify the accuracy of the assembled contigs.

### Phylogenetic studies

The nucleotide sequences and translated amino acid sequences of contigs with high similarity to known viral nucleic acids and proteins in GenBank were used for phylogenetic analysis. Alignments were performed by ClustalW and phylogenetic trees were constructed by the Maximum Likelihood method with a bootstrap of 1,000 replicates through MEGA 6.06 (http://www.megasoftware.net/megamacBeta.php). Gaps were regarded as a pairwise deletion unless specifically noted.

## Results

### Taxonomic identification of ticks

Each tick collected was identified to genus level using a stereomicroscope. Through morphological identification, 80 ticks in NY-11, 135 in NY-13, and 97 in MM-13 groups belonged to the genus *Rhipicephalus*. Molecular identification indicated the presence of multiple species in each group; these included *R*. *microplus*, *R*. *haemaphysaloides*, and *R*. *sanguineus*.

### Ion-torrent sequencing reads

The sequencing run resulted in 4,038,018, 3,835,222, and 4,820,504 reads in tick pools NY-11, NY-13, and MM-13, respectively. After filtering and trimming, there were 3,641,088 reads with a 197-bp average length for NY-11, 3,106,733 reads with a 179-bp average length for NY-13, and 3,871,851 reads with a 180-bp average length for MM-13.

### Viral-related reads in *Rhipicephalus* spp. ticks

**Fig 2:**
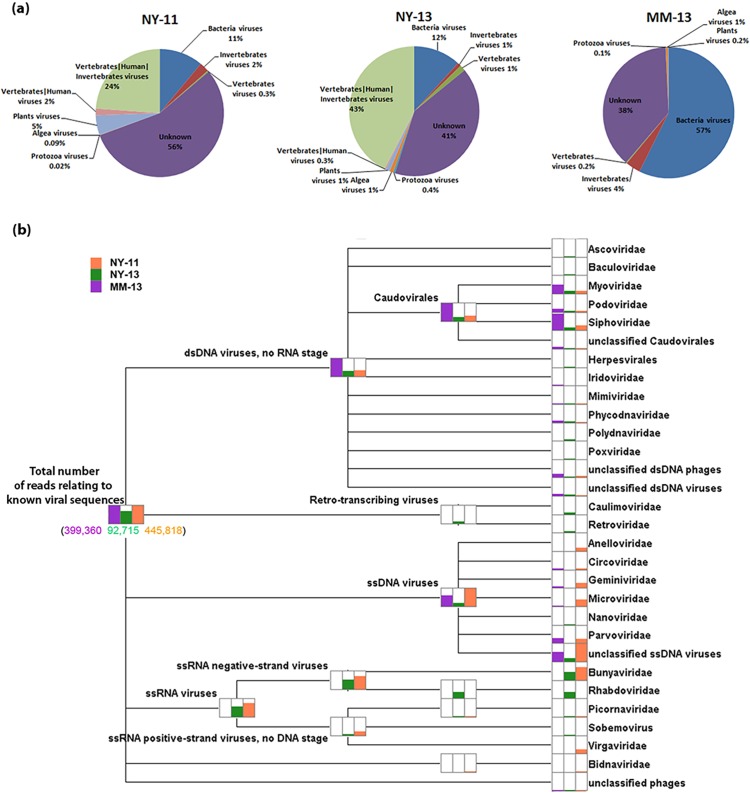
Taxonomic classification overview of metagenomic sequences from the three tick viromes. *(a) Classified by viral host*. Breakdown of the viral sequences into seven categories: vertebrate-human-invertebrate, vertebrate-human, vertebrates, invertebrates, plants, algae, and protozoa viruses. *(b) Classified by viral family*. Viral metagenomic sequences were compared with the viral protein database in GenBank. Comparisons were performed with the BLASTx algorithm using an E-value cutoff 1e-04. The MEGAN5 program (http://ab.inf.uni-tuebingen.de/software/megan5/) was used for acceptance of the output of a BLAST enquiry based on the lowest-common-ancestor algorithm taxonomy analysis.

Based on the most significant BLASTx similarities (e-value<10^−6^), about 12.2% (445,818/3,641,088) of reads from NY-11, 3.0% (92,715/3,106,733) of reads from NY-13, and 10.3% (399,360/3,871,851) of reads from MM-13 were corresponded to known viral sequences.The reads identified from the three tick viromes were classified into bacteria, invertebrates, vertebrates, plants, algae, and protozoan viruses (Fig. 2, S2 Table). In NY-11 and NY-13, the majority of the viral reads classified were vertebrate-human-invertebrate viruses (24% and 43%, respectively), followed by bacterial viruses (11% and 12%, respectively). However, no reads corresponded to vertebrate-human-invertebrate viruses or vertebrate–human viruses in MM-13. In MM-13, most viral-related reads belonged to bacterial viruses (57%). The viral category distribution of NY-11 was similar to NY-13.

From the taxonomic classification overview (Fig. 2, S2 Table), the virus-related reads exist in three viromes distributed across 24 virus families. *Anelloviridae*, *Microviridae*, *Virgaviridae*, *Bidnaviridae* were only found in NY-11, *Ascoviridae*, *Baculovirdae*, *Polydnaviridae*, *Caulimoviridae*, *Retroviridae*, *Nanoviridae*, *Rhabdoviridae* were only found in NY-13, and *Iridoviridae* was only found in MM-13. We found *Bunyaviridae* and *Picornaviridae* in NY-11 and NY-13 and *Circoviridae* and *Parvoviridae* in NY-11 and MM-13. Finally, *Myoviridae*, *Podoviridae*, *Siphoviridae*, *Mimiviridae*, and *Phycodnaviridae* were shared across all three viromes.

### *De novo* consensus assembly of three tick virome reads

The following contigs were assembled from the reads: 1144 contigs from NY-11, 516 contigs from NY-13, and 629 contigs from MM-13. Also, 40, 14 and 64 large contigs (≥1000bp) in three viromes were identified (Table 1). Next, the contigs were aligned against the NCBI nucleotide (nt) and viral genome database using BLASTn; contigs lacking similarity to BLASTn were then aligned against the nr and viral protein database using BLASTx.

**Table 1:** *De novo* assembly of three tick viromes.

**Tick sample**	**No. of reads hit to viral protein database**	**No. of assembled contigs**	**No. of large contigs (>1000bp)**	**% of reads assembled into contigs**	**Average coverage**	**Contig N50 (bases)**
**NY-11**	445,818	1,144	40	67.5 (300,860/445,818)	18	1,274
**NY-13**	92,715	516	14	95.6 (88,627/92,715)	18	843
**MM-13**	399,360	629	64	98.3 (392,580/399,360)	18	1,597

From the BLASTn results (e-value <10^−6^, S3 Table), there were 32 contigs from NY-11 with 79–98%, 27 contigs from NY-13 with 74–99%, and 83 contigs from MM-13 with 72–96% identities to known viral genomic sequences. In the BLASTx alignment results (contigs ≥1000bp, e-value<10^−6^; Table 2), there were 22 contigs from NY-11 with 36–66%, 8 contigs from NY-13 with 39–53%, and 13 contigs from MM-13 with 41–93% sequence identities to known viral protein sequences. Most contigs had low similarity values to known viral proteins, indicating that these sequences may represent novel viruses.

**Table 2:** Large contigs with significant BLASTx similarities to known viruses in three *Rhipicephalus* spp. tick samples.

**Virome**	**ContigNo.**	**Length (bp)**	**BLASTx to NCBI nr and viral protein database**			**Coverage (%)**	**Identity (%)**	**PCR identified**
			**Family**	**Accession No.**	**Best hit (amino acid Start-End, e-value)**			
**NY-11**	240	1,537	*Anelloviridae*	YP_003587834	hypothetical protein gp3 [Torque teno canis virus] (54–491, 1e-108)	85	42.79	Y
	326	1,617	*Bunyaviridae*	NP_950237	nucleoprotein [Crimean-Congo hemorrhagic fever virus] (3–482,5e-125)	89	40.66	Y
	386	1,312	*Bunyaviridae*	NP_690576	L protein [Dugbe virus] (2,874–3,296, 4e-133)	97	48.23	-
	679	1,071	*Bunyaviridae*	NP_950237	nucleoprotein [Crimean-Congo hemorrhagic fever virus] (331–482, 2e-37)	77	51.3	-
	720	1,265	*Bunyaviridae*	NP_950237	nucleoprotein [Crimean-Congo hemorrhagic fever virus] (113–356, 6e-78)	88	36.29	-
	875	2,432	*Bunyaviridae*	YP_325663	putative polyprotein [Crimean-Congo hemorrhagic fever virus] (1,192–1,689, 3e-161)	99	51.95	Y
	1096	2,222	*Bunyaviridae*	NP_690576	L protein [Dugbe virus] (2,863–3,583, 0)	97	48.13	Y
	352	1,760	*Myoviridae*	NP_054647	structural protein [Chlamydia phage 2] (9–565, 0)	89	52.92	-
	848	1,795	*Myoviridae*	YP_022479	structural protein [Chlamydia phage 3] (6–565, 0)	91	54.61	-
	907	1,540	*Myoviridae*	NP_073538	major capsid protein [Bdellovibrio phage phiMH2K] (8–499, 1e-148)	95	48.31	N
	652	1,335	*Siphoviridae*	YP_239849	ORF3 [Staphylococcus phage 2638A] (440–603, 2e-107)	94	63.86	-
	851	1,429	*Siphoviridae*	YP_008058782	DNA polymerase [Staphylococcus phage StauST398-2] (171–516, 3e-159)	99	66.48	-
	894	1,620	*Siphoviridae*	NP_150177	putative anti-receptor protein [Streptococcus phage MM1] (963–1,503, 1e-101)	99	43.41	-
	16	1,480	*unclassified ssDNA viruses*	YP_006908226	major capsid protein [Dragonfly-associated microphage 1] (1–445, 8e-157)	93	53.26	Y
	839	1,397	*unclassified ssDNA viruses*	YP_006908226	major capsid protein [Dragonfly-associated microphage 1] (4–326, 3e-88)	98	42.86	-
	10	1,272	*unclassified ssDNA*	YP_003104795	coat protein [Sclerotinia sclerotiorum hypovirulence associated DNA virus 1] (185–312, 6e-42)	62	59.38	-
	130	1,129	*unclassified ssDNA*	YP_003104795	coat protein [Sclerotinia sclerotiorum hypovirulence associated DNA virus 1] (176–312, 3e-50)	87	43.26	-
	454	1,238	*unclassified ssDNA*	YP_003104795	coat protein [Sclerotinia sclerotiorum hypovirulence associated DNA virus 1] (58–312, 2e-68)	62	44.94	-
	817	1,274	*unclassified ssDNA*	YP_003104795	coat protein [Sclerotinia sclerotiorum hypovirulence associated DNA virus 1] (40–312, 2e-75)	66	47.16	Y
	913	1,166	*unclassified ssDNA*	YP_003104795	coat protein [Sclerotinia sclerotiorum hypovirulence associated DNA virus 1] (108–3, 9e-46)	85	45.18	-
	637	1,580	*unclassified ssDNA*	YP_003104796	replication-associated protein [Sclerotinia sclerotiorum hypovirulence associated DNA virus 1] (6–321, 6e-103)	80	45.71	Y
	846	1,549	*unclassified ssDNA*	YP_003104796	replication-associated protein [Sclerotinia sclerotiorum hypovirulence associated DNA virus 1] (1–224, 3e-68)	67	54.19	-
**NY-13**	3	1,605	*Bunyaviridae*	NP_950237	nucleoprotein [Crimean-Congo hemorrhagic fever virus] (3–482, 4e-125)	90	40.66	Y
	10	1,462	*Bunyaviridae*	NP_690576	L protein [Dugbe virus] (2,233–2,694, 0)	95	58.87	Y
	15	1,188	*Bunyaviridae*	NP_690576	L protein [Dugbe virus] (892–1,246, 1e-96)	90	43.38	-
	28	2,059	*Bunyaviridae*	NP_690576	L protein [Dugbe virus] (3,115–3,649, 0)	99	44.67	-
	31	1,252	*Bunyaviridae*	YP_325663	putative polyprotein [Crimean-Congo hemorrhagic fever virus] (1,285–1,689, 3e-147)	98	54.37	Y
	2	1,007	*Rhabdoviridae*	YP_007641386	large polymerase protein [Isfahan virus] (1,566–1,888, 2e-64)	97	39.63	-
	5	2,860	*Rhabdoviridae*	YP_007641386	large polymerase protein [Isfahan virus] (477–1,422, 0)	99	47.4	Y
	7	1,458	*Rhabdoviridae*	NP_116748	polymerase [Spring viraemia of carp virus] (201–668, 2e-166)	96	53.19	-
**MM-13**	39	2,001	*Podoviridae*	YP_008240601	DNA polymerase [Cellulophaga phage phi4:1] (249–706, 5e-154)	96	53.46	-
	12	1,605	*Myoviridae*	YP_006987664	hypothetical protein GAP52 017 [Cronobacter phage vB CsaP GAP52] (1–430, 2e-148)	80	53.24	-
	484	1,264	*Myoviridae*	YP_007348471	putative dTDP-glucose 4,6-dehydratase [Escherichia phage phAPEC8] (1–326, 1e-104)	89	52.68	-
	42	1,291	*Siphoviridae*	YP_239811	ORF001 [Staphylococcus phage 2638A] (1,361–1,597, 3e-149)	98	81.01	N
	20	1,597	*Siphoviridae*	YP_239726	ORF010 [Staphylococcus phage 85] (6–310, 2e-124)	68	61.11	-
	30	1,621	*Siphoviridae*	YP_008239630	phage structural protein [Salmonella phage FSL SP-031] (19–292, 0)	99	93.07	-
	112	1,010	*Siphoviridae*	YP_008239630	phage structural protein [Salmonella phage FSL SP-031] (17–229, 0)	98	92.96	-
	131	1,245	*Siphoviridae*	YP_008239630	phage structural protein [Salmonella phage FSL SP-031] (2–229, 0)	75	83.19	-
	470	1,418	*Siphoviridae*	YP_008239630	phage structural protein [Salmonella phage FSL SP-031] (20–428, 0)	99	89.24	Y
	5	1,654	*Siphoviridae*	YP_007678083	hypothetical protein [Bacillus phage PM1] (20–357, 7e-81)	88	41.3	N
	8	1,047	*Siphoviridae*	YP_007112079	putative protease/scaffold protein [Enterobacteria phage mEp460] (217–577, 6e-164)	98	63.81	-
	284	1,885	*Siphoviridae*	NP_680517	putative primase [Lactobacillus phage A2] (5–309, 2e-81)	69	44.63	-
	205	1,273	*unclassified ssDNA viruses*	YP_001429872	portal protein [Staphylococcus phage tp310-1] (49–415, 0)	88	86.45	-

For the PCR results, “Y” indicates that the contig was detected in that specific sample while “N” indicates that it was not, “-” indicates the PCR screening hasn’t been conducted.

### Animal and human viruses identified in ticks

Contigs from three tick viromes were similar to those from three families of animal viruses, namely, *Bunyaviridae*, *Anelloviridae*, *and Rhabdoviridae*.

#### *Nairovirus* (*Bunyaviridae* family)

The *Nairovirus* genus contains the following seven serogroups: Crimean-Congo hemorrhagic fever, Dera Ghazi Khan, Hughes, Nairobi sheep disease, Qalyub, Sakhalin, and Thiafora. All of them are tick-borne and contain some of the most important tick-borne viruses [25]. CCHFV, a representative member of the *Nairovirus* genus, has one of the widest geographical distributions of medically important arboviruses and causes severe hemorrhagic fever syndrome in humans with a mortality rate ranging from 30–50% [3]. NSDV, another important virus, is highly pathogenic to sheep and goats and can cause disease in humans [26].

*Nairovirus* has three separate negative-stranded RNA (L, M, and S) which encoding RNA polymerase, pre-glycoprotein and nucleoprotein, respectively [27]. Numerous reads and contigs from NY-11 and NY-13 viromes were found to have statistically significant (e-value < 10^−6^) relationships with *Nairovirus* (Table 2, S3 Table). Contig 326 (from NY-11, Genbank Accession Number: KP141755) and contig 3 (from NY-13, Genbank Accession Number: KP141756) covered 99.9% of the open reading frame (ORF) of the *Nairovirus* nucleoprotein (40.66% identity). The contigs corresponding to the *Nairovirus* L protein cover most of the L protein ORF. Additionally, the cross-blastn results revealed overlapping nucleic acid regions between NY-11 and NY-13 contigs (related to *Nairovirus*) that had almost the same sequences (99.8% identity). The specific primers (S1 Table) based on known contigs related to *Nairovirus* L protein were used to fill the gaps between the contigs, thereby generating a consensus sequence (11435 nt) with 90% coverage of the *Nairovirus* L protein.

**Fig 3:**
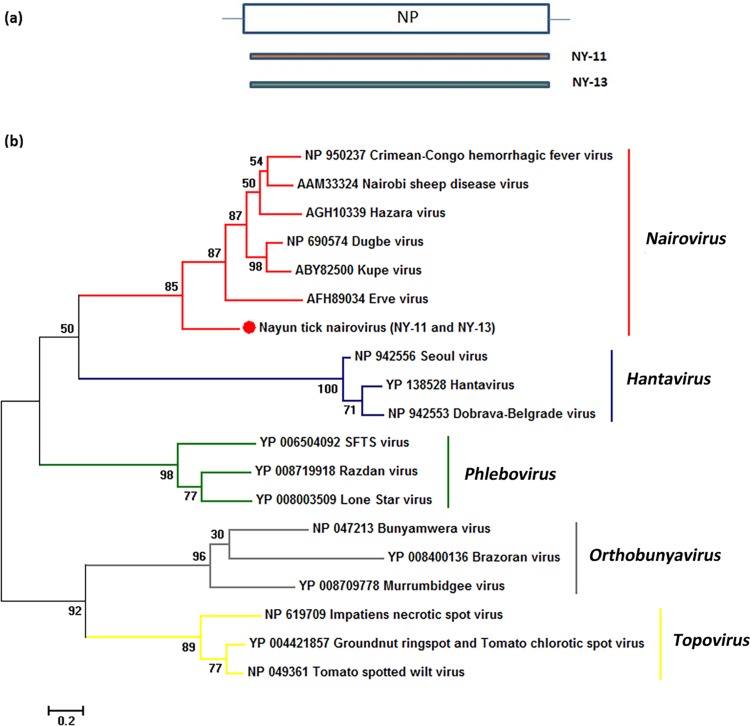
Phylogenetic analysis of nairovirus-related sequences from ticks. (a) Schematic representation of the S segment encoded nucleoprotein (∼480 amino acids) of *Nairovirus*. The orange and green bars represent the contig sequences from NY-11 and NY-13. (b) Phylogenetic analysis of the selected contigs related to *Nairovirus* is based on alignments with 480 amino acids of the nucleoprotein. All five *Bunyaviridae* genera are indicated on the right of the tree. NP denotes nucleoprotein.

The tick nairovirus-related nucleoprotein amino acid sequence was used to determine phylogenetic relationships. The result showed it clustered with the *Nairovirus* genus, but was distantly related to the known nairoviruses (Fig. 3), suggesting that the sequence may represent a novel virus belonging to the *Nairovirus* genus. This virus was named as Nayun tick nairovirus (NTNV).

#### *Thetatorquevirus* (*Anelloviridae* family)

**Fig 4:**
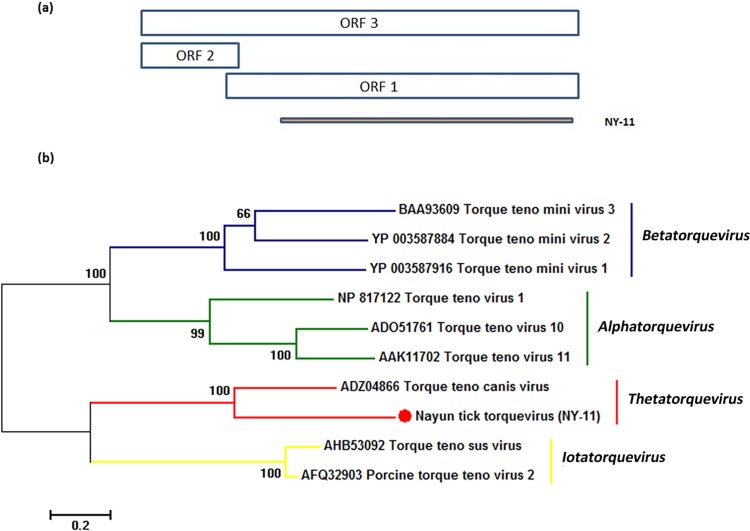
Phylogenetic analysis of tick thetatorquevirus-related sequences. (a) Schematic representation of three ORFs for *Thetatorquevirus*. The orange bar represents the contig sequence from NY-11. (b) Phylogenetic analysis of the selected contig related to *Thetatorquevirus* is based on an alignment with the predicted 440 amino acid sequence of ORF1. *Thetatorquevirus* and three other genera from the *Anelloviridae* family are indicated on the right of the tree.

*Thetatorquevirus*, a recently discovered genus of the *Anelloviridae* family, is a small, non-enveloped DNA virus with a circular single-stranded DNA genome (∼2–3 kb) containing three or four overlapping ORFs (ORF1, ORF2 and ORF3) [28]. The known hosts for anelloviruses include humans, non-human primates and domestic animals. Viruses belonging to this family are usually identified in blood [29], and ticks may obtain viruses from viremic hosts during blood feeding. The top BLASTx hit for NY-11 contig240 (Genbank Accession Number: KP141758) had an amino acid identity of 43% and covered 85% of ORF1 of the Torque teno canis virus (TTcV) (Table 2). Phylogenetic analysis based on the amino acid sequence encoded by ORF1 and the topology of the tree indicated that the contig sequence related to *Anelloviridae* in sample NY-11(Nayun tick torquevirus, NTTV) is a novel virus cluster with TTcV (Fig. 4).

#### *Rhabdovirus* (*Rhabdoviridae* family)

**Fig 5:**
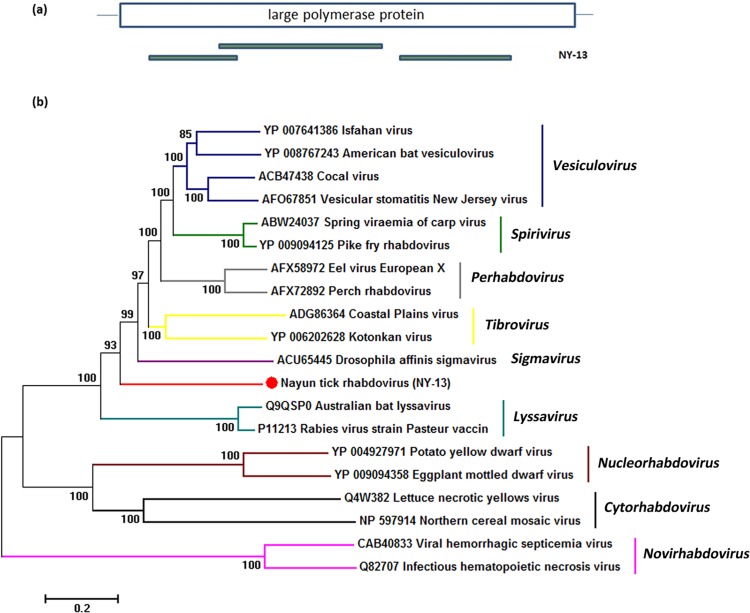
Phylogenetic analysis of tick rhabdovirus-related sequences. (a) Schematic representation of the large polymerase protein of rhabdovirus. The green bar represents the contig sequence from NY-13. (b) Phylogenetic analysis of the selected contig related to rhabdovirus is based on an alignment with an 889 amino acid sequence from the large polymerase protein region of rhabdovirus. All nine *Rhabdoviridae* genera are indicated on the right of the tree.

The family *Rhabdoviridae* family presents in vertebrates, invertebrates and plants. The viral genome is a negative-sense single-stranded RNA containing at least five ORFs that encode its structural protein (N, P, M, G and L). Currently, *Rhabdoviridae* consist of nine named genera [30], and many rhabdoviruses still await taxonomic classification. Rabies virus (genus *Lyssavirus*) is the best-known member of this family which can cause lethal disease of human. Many viruses from genus *Vesiculovirus* are typical arboviruses. There were many reports about rhabdovirus from ticks. Such as Isfahan virus which belongs to the genus *Vesiculovirus* has been isolated from *Hyalomma asiaticm* ticks in Turkmenia [31]. And the other rhabdoviruses isolated from ticks are currently unassigned. Such as Kolente virus which isolated from *Amblyomma variegatum* ticks in the Republic of Guinea [32], and Long island tick rhabdovirus from *Amblyomma americanum* ticks in New York State of American [30]. In sample NY-13, three large contigs (contig 2, 5 and 7) shared amino acid sequence similarity levels of 40–53% with the large polymerase protein (L) of rhabdovirus which belongs to the genus *Vesiculovirus*. A partial sequence from the large polymerase protein (L) region (Genbank Accession Number: KP141757) was used for determining phylogenetic relationships. However the phylogenetic tree revealed that tick rhabdovirus from NY-13 represents a distinct and divergent lineage which shows no clear relationship to *Vesiculovirus* and any other named genus (Fig. 5). This tick unclassified rhabdovirus (Nayun tick rhabdovirus, NTRV) represents a novel species in the family *Rhabdoviridae*.

### Plant viruses

*Tobamovirus* belongs to the family *Virgaviridae*, which comprises 29 definitive and 6 unclassified species. *Tobamovirus* can cause disease in a wide range of hosts, including tobacco, tomato, pepper, orchid, cucumber, melon, bean, and cruciferous plants, resulting in serious economic losses in both field and greenhouse-grown crops. *Tobamovirus* contains positive-sense single-stranded RNA encoding at least four proteins. Tobacco Mosaic Virus (TMV) is the best characterized member of this family, with a genome size of about 6.4 kb in length [33]. We identified 26 contigs in NY-11 sharing high nucleotide sequence similarity scores with *Tobamovirus*. All of them relating to TMV had 89–98% sequence identity scores and comprised ∼52% coverage of the TMV genome.

### Phages

The three tick viromes contained a large diversity of phages, including members from *Myoviridae*, *Podoviridae* and *Siphoviridae* as well as unclassified phages (Fig. 2). In the tick virome of MM-13 particularly, all of the contigs related to these phages (Table 2, S3 Table). Most of the phage sequences found in the ticks only shared amino acid identity with known phages. However, there were 31 contigs with 82–96%, 31 contigs with 74–91%, 13 contigs with 82–96%, and 5 contigs with 84–93% nucleotide sequence identity scores shared with the *Staphylococcus* phage known as Twort, *Salmonella* phage FSL SP-031, *Vibrio* phage pYD38-A, and *Staphylococcus* phage EW (S3 Table). These findings indicated that these closely related phages were present in the MM-13 tick virome.

The phages identified in the tick virome may have originated from the bacterial flora of the tick or that of the hosts they had fed upon. *Staphylococcus* (the host for the *Staphylococcus* phages Twort and EW) and *Salmonella* (the host for the phage FSL SP-031) bacteria have both been reported in ticks previously [34, 35].

### Other viruses

Two large contigs (contigs 16 and 839) in NY-11 share amino acid sequence similarities with the major capsid protein of unclassified ssDNA viruses denoted as Dragonfly-associated microphage 1. This microphage is a single-stranded, circular DNA virus with a 4.5-kb genome encoding five ORFs (replication-associated protein, major capsid protein, two hypothetical proteins and a putative DNA pilot protein). Furthermore, seven contigs in NY-11 shared amino acid sequence similarities with unclassified ssDNA viruses including the fungal virus Sclerotinia sclerotiorum hypovirulence-associated DNA virus 1 (SSHSDV-1) [36]. SSHSDV-1, which infects the fungus *Sclerotinia sclerotiorum*, is a single-stranded DNA virus with a circular genome (∼ 2.2 kb) containing two major ORFs, a 312 amino acid coat protein and a replication-associated protein of 324 amino acids. This virus is the only known example of a DNA virus that infects a fungus. In seven contigs, five of them (contig10, 130, 454, 817, and 913) were related to the coat protein, and two of them (contig637 and 846) to the replication-associated protein of SSHSDV-1.

## Discussion

*Rhipicephalus* spp. tick is well suited as a vector of zoonotic disease because it feeds on a wide assortment of animal hosts in the wild, as well as humans. Moreover, it is a vector for many pathogenic agents, such as CCHFV, NSDV, Chim virus, Thogoto virus, and Kandam virus [1, 37, 38]. Furthermore, the geographic distribution of *Rhipicephalus* spp. is extensive in southwest China. Therefore, it is important to use highly sensitivity methods to identify or monitor medically important viruses in *Rhipicephalus* spp. The present study is the first where NGS was used to explore the variety of viral communities that exist in *Rhipicephalus* spp. ticks in China. High-throughput sequencing was performed by the Ion-torrent technique (Invitrogen). It can result in higher speed, lower cost, and smaller instrument size. Compared to the Hiseq 2000 technique, the sequencing quality of Ion Torrent is more stable, has a higher map rate, and the GC depth distribution is better [39]. The Ion 318 chip we used can get > 1 GB data in 2 hours, and the maximum reads length is ∼ 400 bp.

Through BLASTx and BLASTn analyses, a large number of viral sequences were found in ticks, some of which had a close relationship with known viral sequences, while some additional sequences potentially corresponded to novel viruses.

By comparing the three tick viromes, the most abundant sequences were from animal viruses (especially those related to *Nairovirus*), followed by phages in NY-11 and NY-13. However, the most abundant sequences in MM-13 were phages, followed by invertebrate viruses, while there were only a few sequences corresponding to animal viruses (Fig. 2, S2 Table).

When we compared the virome of MM-13 with those of NY-11 and NY-13, only some bacteriophages were commonly shared. The distance between the tick sampling sites at Nayun and Mengma was about 15–20 km; however, the viral communities differed extensively at these two sites, possibly indicating that ticks in these two regions have separate natural ecological environments. Or this could be a result of individual differences in the pooled samples.

Almost all known nairoviruses are transmitted by ticks, and some of them, such as CCHFV and NSDV, have been isolated from or identified in *Rhipicephalus* spp.; these viruses can cause serious diseases in humans and livestock [1]. In the present study, a new tick nairovirus was identified in Nayun (NY-11 and NY-13) through deep sequencing, and then confirmed by specific PCR amplification. For the assembled contigs from NY-11 and NY-13, many contigs shared amino acid similarity (∼40%) with the S and L protein of *Nairovirus*. Additionally, cross-BLASTn showed very high similarity (> 98% nucleic acid identity) scores between the contigs relating to *Nairovirus* from NY-11 and NY-13. These findings indicated that the same nairovirus-related agent was present in the NY-11 and NY-13 tick samples. The phylogenetic analysis indicated that the NTNV clustered with *Nairovirus*, but had a distant relationship with known nairoviruses. Unfortunately, we did not obtain the large, assembled contigs corresponding to the *Nairovirus* M protein (glycoprotein). The lack of recognizable M segment is consistent with the reports about the tick virome in the U.S. [17]. They recovered > 90% of the L and S segments for one nairovirus and two phleboviruses, however the sequences with similarity to *Bunyavirdae* M segments were unable to identify. This may be due to the complicated secondary structure of M segments which inhibit efficient cDNA synthesis and interfere with amplification. Or because the glycoprotein has greater variation than nucleoprotein and L polymerase, we can’t get the related sequences through the alignment with the known database.

Our analysis uncovered the anellovirus and phages in ticks, which is very similar to what’s found and proposed in mosquito [16]. The anelloviurs related sequences in tick virome were novel, which suggested that the animal hosts the ticks feed on contain uncharacterized anellovirus. Like the phages identified in mosquitoes, the tick viromes contained a large diversity of phages sequences (Fig. 2 and Table 2, S2 Table). It is possible that the tick acquires phages during blood feeding, or it is possible phages originate from the tick.

Additionally all three tick viromes contained some sequences related to the following plant viruses: *Caulimoviridae*, *Nanoviridae*, *Geminiviridae*, *Virgaviridae* and *Sobemovirus* (Fig. 2, S2 Table); in the tick virome study in the U.S., they found plant related viruses which belong to genus *Sobemovirus* [17].

In conclusion, this study revealed the presence of highly novel and diverse viral communities in ticks. This information will provide better understanding of the virome via knowledge about the presence and transmission of disease-causing viruses in ticks under natural conditions.

## Supporting Information

S1 Table List of primers used in this study. (DOC)

S2 Table Reads related viral family and genus. Table shows the viral genus, family and host information of the reads from three tick viromes.
(DOCX)

S3 Table Analysis of the contigs with nucleotide identities (BLASTn, e-value < 10^−6^) to known viruses. (DOC)
