# Supplementary material for: Metagenomic Profile of the Viral Communities in Rhipicephalus spp. Ticks from Yunnan, China
Source: PLoS One. 2015 Mar 23;10(3):e0121609. doi: 10.1371/journal.pone.0121609 (PMC4370414; doi:10.1371/journal.pone.0121609)
Supplement: S1 Table — (DOC) [file pone.0121609.s001.doc]

**Table S1. List of primers used in this study**

| **Primers name** | **5’-3’** |
| --- | --- |
| **For tick identification** |  |
| ITS-1F | TCATAAGCTCGCGTTGATT |
| ITS-1R | AGCTGGCTGCGTTCTTCAT |
| ITS-2F | CGAGACTTGGTGTGAATTGCA |
| ITS-2R | TCCCATACACCACATTTCCC |
| **For cDNA library (Brs)** |  |
| NY-11 | CATCACATAGGCGTCCGCTGNNNNNNNNN |
| NY-13 | ACGGTGTGTTACCGACGTCCNNNNNNNNN |
| MM-13 | CCGAGGTTCAAGCGAGGTTGNNNNNNNNN |
| **For selected contigs** |  |
| NY-11 Contig 240 | CAAAAGGGAACAGGAAATGCTTAC |
|  | CCCATAGCTGCTATTCTCAAACATG |
| NY-11 Contig 326 | TCGGCTTCCACATCAATGACTTC |
|  | CTGGGCACACTGTGCAAAGTACC |
| NY-11 Contig 875 | TCTATCAGTTCGCAAAAGGTACGC |
|  | TGTTGCATGACATGCACTACAGTG |
| NY-11 Contig 1096 | ATGGGCTAGCTAAGTGCACCCTGG |
|  | AATTCTCTCATACCGCCTACTAGT |
| NY-11 Contig 907 | GCACAAAACCACCTTCAACGGCG |
|  | GTAACTAATGGATGTGTCTGGA |
| NY-11 Contig 16 | AGTTAATACCATGTTCACTTAT |
|  | GACCAACATTAGTAGTCGTGTC |
| NY-11 Contig 817 | GAAATGACATGCTAACGTACTC |
|  | ACTCGAAGACGCCGGTCTTATTA |
| NY-11 Contig 637 | CGTTAACGACCATTTTGCATCTC |
|  | CGGTACCATCACCAGAACTGAATC |
| NY-13 Contig 3 | AGGCAGTGACCCTGGGCTAAA |
|  | GCCTCTGCCGCTGGATTCTTG |
| NY-13 Contig 10 | GCTTCCAATCCCACTGATGTAG |
|  | GCCAAGAGACAGCGACTGAACA |
| NY-13 Contig 31 | CTACTGCGACTTAACTCCACTC |
|  | ACCTGAAAACACTGTTGTGAAGT |
| NY-13 Contig 5 | ACGTGACTTTCTAAAGCTGGTT |
|  | TAAAGCTTTGTTCAAAAAACTCA |
| MM-13 Contig 42 | ACGGTGGCTATCTATTCGATTAT |
|  | CACGACCGCCTCCAGAGCTTTTCA |
| MM-13 Contig 470 | GATTGCGTTGAGTATAAAGGAAA |
|  | AAACGCTACCTTATCTCCCAACT |
| MM-13 Contig 5 | AGTGCGGCGACCTTGATCCATG |
|  | CCCCATGCAGCCAGGTCAGGTAC |
| **For Nairovirus L segment gap filled** |  |
| LORF-gap1F | TTGATCCGTCTGTGCATGGAACTAT |
| LORF-gap1R | CAGTCCTAGTGTGCTCAGAATACCC |
| LORF-gap2F | CAGGAAACAGTATTTTATGGCAAGC |
| LORF-gap2R | TTTCCTTACGAATGTCTTTTAGGATT |
| LORF-gap3F | TTCTATCCAGACCTTATGAGGCATG |
| LORF-gap3R | ATTGCTGTTATGCAGTGCTGTACCT |
| LORF-gap4F | CTGATGAGTTTCCTCAACTGGAAGC |
| LORF-gap4R | GACTCACTACAGTATGTGAACCGGC |
| LORF-gap5F | CGGAGAGGGGCAACTTTTCA |
| LORF-gap6R | ATGCTTTGCACTTGCCTCCT |
