# Supplementary material for: Metagenomic Profile of the Viral Communities in Rhipicephalus spp. Ticks from Yunnan, China
Source: PLoS One. 2015 Mar 23;10(3):e0121609. doi: 10.1371/journal.pone.0121609 (PMC4370414; doi:10.1371/journal.pone.0121609)
Supplement: S2 Table — Table shows the viral genus, family and host information of the reads from three tick viromes. (DOCX) [file pone.0121609.s002.docx]

Table S2. Reads related viral family and genus

| **Family and genus** | **Host** | **Tick pool** | | |
| --- | --- | --- | --- | --- |
|  |  | **NY-11** | **NY-13** | **MM-13** |
| ***Ascoviridae*** |  |  |  |  |
| *Ascovirus* | Invertebrates | 0 | 134 | 0 |
| ***Baculoviridae*** |  |  |  |  |
| *Alphabaculovirus* | Invertebrates | 0 | 50 | 0 |
| *other baculovirus* | Invertebrates | 0 | 59 | 0 |
| ***Myoviridae*** |  |  |  |  |
| *I3likevirus* | Bacteria | 0 | 188 | 0 |
| *Bcepmulikevirus* | Bacteria | 0 | 0 | 51 |
| *Felixounalikevirus* | Bacteria | 0 | 0 | 57 |
| *P2likeviruses* | Bacteria | 963 | 153 | 0 |
| *Phikzlikevirus* | Bacteria | 0 | 91 | 0 |
| *Punalikevirus* | Bacteria | 0 | 81 | 0 |
| *Spounalikevirus* | Bacteria | 0 | 0 | 65 |
| *Twortlikevirus* | Bacteria | 226 | 392 | 23,024 |
| *T4likevirus* | Bacteria | 70 | 330 | 395 |
| *Viunalikevirus* | Bacteria | 0 | 0 | 75 |
| *other myoviruses* | Bacteria | 3,100 | 3,112 | 22,932 |
| ***Podoviridae*** |  |  |  |  |
| *N4likevirus* | Bacteria | 0 | 0 | 2,865 |
| *other podoviruses* | Bacteria | 693 | 801 | 3,600 |
| ***Siphoviridae*** |  |  |  |  |
| *Lambdalikevirus* | Bacteria | 1,719 | 297 | 517 |
| *T5likevirus* | Bacteria | 0 | 0 | 109 |
| *Yualikevirus* | Bacteria | 0 | 0 | 267 |
| *other siphoviruses* | Bacteria | 10,485 | 5,092 | 167,383 |
| ***Iridoviridae*** |  |  |  |  |
| *Lymphocystivirus* | vertebrates | 0 | 0 | 162 |
| *other iridoviruses* | ? | 0 | 0 | 10 |
| ***Mimiviridae*** |  |  |  |  |
| *Mimivirus* | Protozoa | 0 | 330 | 0 |
| *other mimiviruses* | Protozoa | 97 | 82 | 463 |
| ***Phycodnaviridae*** |  |  |  |  |
| *Chlorovirus* | Algae | 242 | 462 | 1,666 |
| *Phaeovirus* | Algae | 0 | 58 | 0 |
| *Prasinovirus* | Algae | 0 | 135 | 69 |
| *Prymnesiovirus* | Algae | 71 | 164 | 228 |
| *other phycodnaviruses* | Algae | 95 | 85 | 86 |
| ***Polydnaviridae*** |  |  |  |  |
| *Bracovirus* | Invertebrates | 0 | 620 | 0 |
| ***Poxviridae*** |  |  |  |  |
| *Avipoxvirus* | Vertebrates | 0 | 228 | 0 |
| *other poxviruses* | ? | 0 | 44 | 0 |
| ***Caulimoviridae*** |  |  |  |  |
| *Badnavirus* | Plants | 0 | 542 | 0 |
| *Caulimovirus* | Plants | 0 | 267 | 0 |
| *Soymovirus* | Plants | 0 | 74 | 0 |
| ***Retroviridae*** |  |  |  |  |
| *Gammaretrovirus* | Vertebrates | 0 | 275 | 0 |
| *Spumavirus* | Vertebrates | 0 | 215 | 0 |
| *other retroviruses* | Vertebrates | 0 | 77 | 0 |
| ***Nanoviridae*** | Plants | 0 | 61 | 0 |
| ***Anelloviridae*** |  |  |  |  |
| *Thetatorquevirus* | Vertebrates\| Human | 7,507 | 0 | 0 |
| ***Circoviridae*** |  |  |  |  |
| *Circovirus* | Vertebrates | 1,158 | 0 | 805 |
| *other circoviruses* | Vertebrates | 348 | 0 | 7 |
| ***Geminiviridae*** |  |  |  |  |
| *Begomovirus* | Plants | 8,852 | 0 | 501 |
| *Mastrevirus* | Plants | 530 | 0 | 60 |
| *other geminiviruses* | Plants | 3,382 | 0 | 47 |
| ***Microviridae*** |  |  |  |  |
| *Bdellomicrovirus* | Bacteria | 2,015 | 0 | 0 |
| *Chlamydiamicrovirus* | Bacteria | 19,005 | 0 | 0 |
| *Spiromicrovirus* | Bacteria | 1,049 | 0 | 0 |
| *other microviruses* | Bacteria | 7,987 | 0 | 72 |
| ***Parvoviridae*** |  |  |  |  |
| *Densovirus* | Invertebrates | 149 | 0 | 0 |
| *Iteravirus* | Invertebrates | 3,823 | 0 | 945 |
| *Pefudensovirus* | Invertebrates | 673 | 0 | 3,126 |
| *other parvoviruses* | ? | 6,134 | 0 | 10,623 |
| ***Bunyaviridae*** |  |  |  |  |
| *Nairovirus* | Vertebrates\| Invertebrates\| Human | 106,392 | 38,996 | 0 |
| *Phlebovirus* | Vertebrates\| Invertebrates\| Human | 0 | 364 | 0 |
| ***Rhabdoviridae*** |  |  |  |  |
| *Ephemerovirus* | ? | 0 | 234 | 0 |
| *Lyssavirus* | Vertebrates\| Human | 0 | 283 | 0 |
| *Perhabdovirus* | ? | 0 | 498 | 0 |
| *Sigmavirus* | ? | 0 | 747 | 0 |
| *Siniperca chuatsi rhabdovirus* | Vertebrates | 0 | 191 | 0 |
| *Vesiculovirus* | ? | 0 | 1,480 | 0 |
| *other rhabdoviruses* | ? | 0 | 18,975 | 0 |
| ***Picornaviridae*** |  |  |  |  |
| *Enterovirus* | Vertebrates | 110 | 308 | 0 |
| ***Virgaviridae*** |  |  |  |  |
| *Tobamovirus* | Plants | 9,312 | 0 | 0 |
| ***Bidnaviridae*** |  |  |  |  |
| *Bidensovirus* | ? | 169 | 0 | 0 |
|  |  |  |  |  |
| ***Sobemovirus*** | Plants | 0 | 182 | 0 |
| ***Herpesvirales*** |  | 0 | 59 | 0 |
| ***unclassified phages*** | Bacteria | 986 | 303 | 7527 |
| ***unclassified dsDNA viruses*** | ? | 255 | 693 | 2175 |
| ***unclassified ssDNA viruses*** | ? | 223420 | 6801 | 57571 |
