# Supplementary material for: Metagenomic Profile of the Viral Communities in Rhipicephalus spp. Ticks from Yunnan, China
Source: PLoS One. 2015 Mar 23;10(3):e0121609. doi: 10.1371/journal.pone.0121609 (PMC4370414; doi:10.1371/journal.pone.0121609)
Supplement: S3 Table — (DOC) [file pone.0121609.s003.doc]

**Table S3. Analysis of the contigs with nucleotide identities (BLASTn, e-value < 10^-6^) to known viruses**

| **Blastn** |  | **NY-11** |  |  | **NY-13** |  |  | **MM-13** |  |  |
| --- | --- | --- | --- | --- | --- | --- | --- | --- | --- | --- |
| **Best hit (e-value<10^-6^)** | **Family** | **No. of contigs** | **Min.% n.t.**  **identity** | **Max.% n.t. identity** | **No. of contigs** | **Min.% n.t.**  **identity** | **Max.% n.t. identity** | **No. of contigs** | **Min.% n.t.**  **identity** | **Max.% n.t. identity** |
| **Animal and Human virus** |  |  |  |  |  |  |  |  |  |  |
| Crimean-Congo hemorrhagic fever virus | *Bunyaviridae* | 1 | 85.34 | 85.34 | 1 | 99 | 99 |  |  |  |
| Dugbe virus | *Bunyaviridae* | 3 | 75.54 | 87.1 | 12 | 74.46 | 87.3 |  |  |  |
| **Becteria virus** |  |  |  |  |  |  |  |  |  |  |
| Staphylococcus phage Twort | *Myoviridae* |  |  |  | 2 | 88.92 | 90.46 | 31 | 82.29 | 96.31 |
| Enterobacteria phage P4 | *Myoviridae* |  |  |  | 1 | 91.63 | 91.63 |  |  |  |
| Enterobacteria phage vB_KleM-RaK2 | *Myoviridae* |  |  |  |  |  |  | 1 | 72.89 | 72.89 |
| Shigella phage SfIV | *Myoviridae* |  |  |  | 3 | 81.95 | 100 |  |  |  |
| Rhodococcus phage REQ1 | *Siphoviridae* |  |  |  | 1 | 78.47 | 78.47 |  |  |  |
| Caulobacter phage CcrRogue | *Siphoviridae* |  |  |  | 1 | 81.94 | 81.94 |  |  |  |
| Salmonella phage FS LSP-031 | *Siphoviridae* |  |  |  |  |  |  | 31 | 74.6 | 90.64 |
| Staphylococcus phage 2638A | *Siphoviridae* |  |  |  |  |  |  | 1 | 76.66 | 76.66 |
| Staphylococcus phage 42e | *Siphoviridae* | 1 | 87.12 | 87.12 |  |  |  |  |  |  |
| Staphylococcus prophage phi13 | *Siphoviridae* |  |  |  | 1 | 76.01 | 76.01 |  |  |  |
| Staphylococcus phage EW | *Siphoviridae* |  |  |  | 4 | 75.08 | 90.45 | 5 | 84.26 | 93.14 |
| Staphylococcus phage SAP-26 | *Siphoviridae* |  |  |  |  |  |  | 1 | 81.36 | 81.36 |
| Staphylococcus phage vB SepiS-phiIPLA5 | *Siphoviridae* |  |  |  | 1 | 86.47 | 86.47 |  |  |  |
| Staphylococcus prophage tp310-1 | *unclassified* | 1 | 79.31 | 79.31 |  |  |  |  |  |  |
| Vibrio phage pYD38-A | *unclassified* |  |  |  |  |  |  | 13 | 81.89 | 96.09 |
| **Plant virus** |  |  |  |  |  |  |  |  |  |  |
| Tobacco mosaic virus | *Virgaviridae* | 26 | 89.29 | 98.1 |  |  |  |  |  |  |
